# Illusory inferences in conditional expressions

**DOI:** 10.3758/s13421-024-01571-2

**Published:** 2024-04-30

**Authors:** Orlando Espino, Isabel Orenes, Sergio Moreno-Ríos

**Affiliations:** 1 Department of Cognitive, Social, and Organizational Psychology, University of La Laguna, Tenerife, Spain; 2 Department of Basic Psychology, National Distance Education University (UNED), Madrid, Spain; 3 Department of Developmental and Educational Psychology & CIMCYC, University of Granada, Granada, Spain

**Keywords:** Illusory inferences, Conditionals, Mental models

## Abstract

A robber points a gun at a cashier and says: “Only one of these two options is true: If you conceal the combination to the safe, then I kill you; otherwise, if you don´t conceal the combination to the safe, then I kill you.” Hearing this statement, most people conclude that, in either case, “I kill you.” This is an illusory response, in fact; the valid conclusion states “I don´t kill you.” The research reported here studied the roles that different expressions of conditionals (“if-then,” “only if,” and “if and only if”) play in the illusory response. Three experiments show that participants inferred the conclusion “I kill you” from the conditional “if-then” and “I may or may not kill you” from the conditional “only if,” while selecting both options with similar frequency for the biconditional “if and only if.” These results shed light on the main theories of deductive reasoning.

## Introduction

Imagine the following situation: a robber points a gun at a cashier. The robber is a logician. He is aware that cameras are recording. Therefore, he must exercise caution in speaking the truth, to ensure that he does not incriminate himself and potentially jeopardize his position in any future legal proceedings. Finally, he says: “Only one of these two options is true: If you conceal the combination to the safe, then I kill you; otherwise, if you do not conceal the combination to the safe, then I kill you.”

No witness would predict a happy ending for the cashier, except for those who have training in logic. In fact, the only valid conclusion is that the robber does *not* kill the cashier. This is because there are only two options: either (1) the first conditional is true and the second is false, or (2) the first conditional is false and the second is true. As a result, one of the two conditions must be false. In formal logic, the material implication asserts that a conditional is false only when the antecedent is true and the consequent is false. In the previous example, (1) the second conditional is false when the cashier does not conceal (or reveal) the combination and *the robber does not kill him*, and (2) the first conditional is false only when the cashier conceals the combination, and *the robber does not kill him*. Therefore, the two possibilities have one thing in common: the robber does not kill the cashier. There is no possibility in which the robber kills the casher when one conditional is false and the other is true.

Conclusions such as “the robber kills the cashier” are called *illusions* (e.g., Johnson-Laird & Savary, 1996). Illusion is illusory, not simply because people get the wrong answer, but because it is systematic, and people are confident and pervasive in their responses (Khemlani & Johnson-Laird, 2017). The study of illusions in cognitive psychology has helped reveal how people think and test the predictions of theories (Pohl, 2022).

In this research, we aim to elucidate alternative explanations for this cognitive error by testing different conditional expressions. The term *illusion* in cognitive psychology has been known for more than two decades and is found to occur in reasoning based on sentential connectives such as “if-then” and “or” (Johnson-Laird & Savary, 1999; Khemlani & Johnson-Laird, 2009), quantifiers such as “all the artists” and “some of the artists” (Yang & Johnson-Laird, 2000), deontic relations such as “permitted” and “obligated” (Bucciarelli & Johnson-Laird, 2005), causal relations such as “causes” and “allows” (Goldvarg & Johnson-Laird, 2000), assessments of whether sets of assertions are consistent (Johnson-Laird et al., 2004), spatial relations (Mackiewicz & Johnson-Laird, 2012; Ragni et al., 2016), and probabilities (Johnson-Laird & Savary, 1996). Most research has employed arbitrary content to avoid potential pragmatic effects in illusory problems.

Let us present an equivalent structural example of the abovementioned case to show what the phenomenon of illusions is all about in the case of the conditional “if-then” (Illusory Example 1):
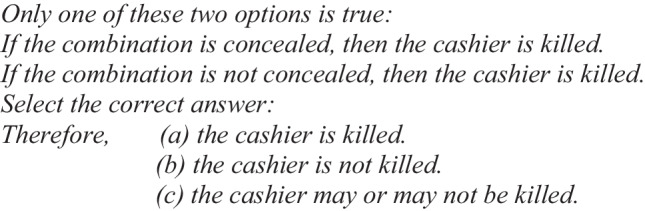


When people solved this type of problem, they chose option (a), “the cashier is killed,” more frequently than options (b) or (c) (Johnson-Laird & Savary, 1999, Experiment 2). However, as we have seen previously, the correct answer is (b), “the cashier is not killed.”

Why, then, do illusions in conditional reasoning occur? Two of the main current theories of reasoning, the suppositional theory (Handley et al., 2006) and the theory of mental models (the “model theory” for short; Khemlani & Johnson-Laird, 2009, 2017) offer different reasons for why people generate such responses in conditional reasoning.

This research has two objectives: the first is to study the role that different conditional expressions play in illusory responses, and the second is to contrast the predictions of the model theory against the suppositional theory about the illusory response. In what follows, we describe how these theoretical approaches explain why illusory responses occur and what their predictions would be for cases of conditional (“if-then” and “only if”) and biconditional (“if and only if”) reasoning. We then describe three experiments that tested these predictions. Finally, we discuss the implications of the results for each of the two main theoretical approaches.

### The suppositional theory

The suppositional theory is one of the several reasoning theories (Fugard et al., 2011; Handley et al., 2006; Oaksford & Chater, 2007; Oberauer & Wilhelm, 2003) that use the probability theory as a rationality framework for human inferences. According to this theory (Evans & Over, 2004; Evans et al., 2005), people evaluate conditionals (such as, “if p then q” or “p only if q”), and biconditionals (“if and only if p, q”) by means of the Ramsey test. That is, they “hypothetically add *p* to their stock of knowledge and evaluate their degree of belief in *q* given *p”* (Ramsey, 1929/1990, p. 155). Thus, they first estimate the probability of the consequent and antecedent occurring together before calculating the probability of the antecedent occurring with the non-occurrence of the consequent. This theory argues that people think only about the possibilities that include the true antecedent, not those that include the false antecedent.

Two important points have been proposed to explain the conclusions given to illusory problems. First, “a false conditional (such as, if p then q) does not imply the presence of p and not-q; it instead means that q does not hold under the supposition of p, and therefore implies ‘if p then not-q.’ The other important point is that not-p cases are irrelevant to determining the truth of a conditional rule” (Handley et al., 2006, p. 568). Given these assumptions, when the previous illusory Example 1, “if-then,” is presented, people tend to select the option “the cashier is killed” over the logically correct option “the cashier is not killed.” From the suppositional theory, there is no illusion in this conclusion. When people solve this problem, they fail to apply the exclusive disjunction between the conditional premises (only one premise is true). The conclusion that people draw from this illusory problem is explained by this theory in the following way (Handley et al., 2006):

People can consider four possibilities:

1. Suppose that “*the combination is concealed, and the cashier is killed”*; then Conditional 1 is true, and since the combination is concealed, Conditional 2 (which states that the combination is *not* concealed) is not applied. Given that the condition that one of the rules is true has been met, the conclusion “the cashier is killed” holds.
2. A similar explanation occurs whenever people suppose that “*the combination is not concealed, and the cashier is killed”*; then Conditional 2 is true, and Conditional 1 is not applied. Again, the condition that one of the rules is true is met, so the conclusion “the cashier is killed” holds.
3. Suppose that “*the combination is concealed, and the cashier is not killed”*; then Conditional 1 is false, and as the combination is concealed, Conditional 2 is not applied. The condition that one of the rules is true is not met, so this possibility can be discounted. The disjunction as a whole is not true.
4. Suppose that *“the combination is not concealed, and the cashier is not killed*”; then Conditional 2 is false, and Conditional 1 is not applied. As in Case 3, the condition that one of the rules is true is not met, so this possibility can be discounted.

According to the suppositional theory, what was previously labeled as an illusory problem is not illusory for participants. This problem arises from a disjunction, where either the first condition is true and the second one is false, or vice versa. Participants can consider four potential scenarios, two of which lead to the conclusion “the cashier is killed,” while the remaining two scenarios are not compatible with the disjunctive problem because a conditional only is true when the antecedent is true.

Regarding the use of other conditionals such as “only if” and “if and only if,” the theory has not made explicit predictions for the illusory problems. However, we do not find reasons to expect different results when the illusory problems are expressed with these other conditionals. Therefore, we will tentatively attribute to the suppositional theory no differences for these expressions.

In sum, the preferred response to the three types of illusory problems (“if-then,” “only if,” and “if and only if”) should be option (a), in which “the cashier is killed.” Table 1 presents the predictions that the suppositional and model theories make for these three illusory problems used in the three experiments of this research.

**Table 1:** Responses predicted based on the suppositional theory and the model theory for illusory problems “if-then,” “only if,” “if and only if”

	Suppositional theory	Model theory
“If-then”	The cashier is killed	The cashier is killed
“Only if”	The cashier is killed	The cashier may or may not be killed
“If and only if”	The cashier is killed	The cashier is killed

### The model theory

The model theory points out that individuals use the meaning of words, the grammatical structure of sentences, and their knowledge of them to construct mental models that simulate the world (Byrne, 2005; Byrne & Johnson-Laird 2019; Johnson-Laird, 1983, 2006; Johnson-Laird & Byrne, 1991; Khemlani et al., 2018). Mental models are *iconic*, that is, their structure corresponds to the structure of what they represent. According to the principle of truth, mental models represent only those possibilities in which an assertion is true (Johnson-Laird & Ragni, 2019). For instance, people may understand “if the combination is concealed, then the cashier is killed” by thinking about the true possibilities: “it is possible that *the combination is concealed and the cashier is killed*”; “it is possible that *the combination is not concealed and the cashier is not killed*”; and “it is possible that *the combination is not concealed and the cashier is killed*.” The “true possibilities” represent situations that render the conditional true, and the only situation in which a conditional is false is “*the combination is concealed, and the cashier is not killed,”* making it a “false possibility” (Johnson-Laird & Byrne, 2002; Khemlani et al., 2018).

The model theory postulates that mental models are parsimonious. People tend to “represent what is possible, but not what is impossible, according to assertions. This principle of parsimony minimizes the load on working memory, and so it applies unless something exceptional occurs to overrule it” (Johnson-Laird, 2006, p. 34). The principle of parsimony is subtle because it applies at two levels. At the first level, people represent only what is mentioned. For example, for a basic conditional such as “if the combination is concealed, then the cashier is killed,” people normally construct a mental model in which the conditional’s antecedent (the combination is concealed) and its consequent (the cashier is killed) are both true, and implicit models (as shown by the ellipsis) that represent the other possibilities in which the antecedent is false:
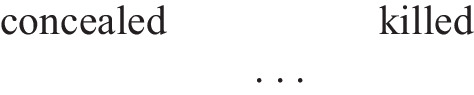


Here, “concealed” stands for “the combination is concealed” and “killed” stands for “the cashier is killed.” At the second level, people also represent implicit models (Johnson-Laird, 2006). They can flesh out one or the two additional possibilities and make them fully explicit, such as:
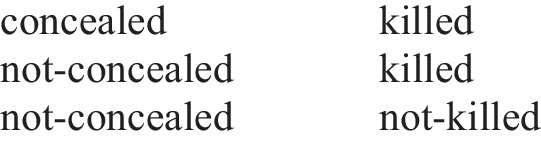


This processing depends on motivation, working memory span, and semantic and pragmatic factors (Johnson-Laird & Byrne, 2002; Johnson-Laird et al., 1992). In sum, from the model theory, people commit illusions in reasoning due to principles of truth and parsimony (Johnson-Laird 2006, 2021; Khemlani & Johnson-Laird, 2009, 2017). In the above Illusory Example 1 (for the conditional “if-then”), people represent this problem with the following initial models (Johnson-Laird & Savary, 1999):
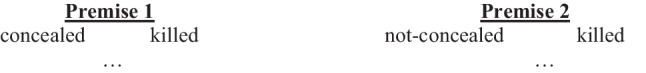


These mental models (or initial models) would lead people to select erroneously the option (a) “The cashier is killed,” since this possibility is represented in both premises. To reach the correct conclusion, people need to flesh out the fully explicit models for both premises:
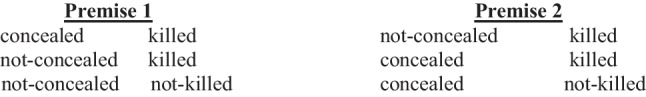


Both premises are consistent with the possibility “the cashier is killed” (“the combination is concealed, and the cashier is killed” and “the combination is not concealed, and the cashier is killed”); however, only one premise can be true for this problem. If Premise 1 is true and Premise 2 is false, then that refers to the possibility “the combination is not concealed, and the cashier is not killed,” whereas if Premise 2 is true and Premise 1 is false, that refers to the possibility “the combination is concealed, and the cashier is not killed.” In either case, the conclusion is “the cashier is not killed.”

Illusions persist for many different types of conditional expression, such as “if-then,” “only if,” and “if and only if.” But the model theory predicts qualitative differences between these conditionals. For some conditionals, people should think about two possibilities; this is known as the “dual principle” (Byrne, 2005; Espino & Byrne, 2013; Espino & Santamaría, 2008; Johnson-Laird & Byrne, 1989; Moreno-Ríos et al., 2008; Santamaría & Espino, 2002; Thompson & Byrne, 2002). Logicians have identified the logical equivalence between the conditional “A only if C” and the conditional “if A, then C.” This equivalence can be illustrated by the observation that the one situation that renders both conditionals false is the one in which the antecedent is true, and the consequent is false (“A and not C”; Jeffreys, 1981). However, several psychological studies have shown that people interpret “A only if C” to mean something subtly different from “if A, then C” (e.g., Cheng & Holyoak, 1985; Evans, 1977; Johnson-Laird & Byrne, 1989). First, evidence from the inferences that participants make suggests that they envisage two initial possibilities from the outset for “A only if C,” i.e., “A and C” and “not-A and not-C*”* (Johnson-Laird & Byrne, 1989). The representation of the two possibilities explains the disappearance of difference in the difficulty between *modus ponens* (MP) and *modus tollens* (MT) for “only if” statements (Girotto et al., 1997; Johnson-Laird & Byrne, 1991) and the greater frequency of MT inferences from “only if” compared to “if-then” (Byrne, 2005). Second, evidence from reading times suggests that people envisage two initial possibilities for “A only if C” statements (Santamaria & Espino, 2002). These authors found that people grasp the possibility “not-A and not-C*”* more quickly when stated using an “only if” rather than an “if-then” conditional. Third, experimental evidence indicates that people envisage the elements of the statement in the direction opposite to the order of mention (from the consequent to the antecedent; Egan et al., 2009; Evans, 1993). However, given that the order is not relevant in this research, for clarity and comparability we will maintain the order in the models as in the propositions. What is important is that regardless of the order in which the antecedent and consequent are represented, the model theory predicts that “only if” gives rise to two possibilities while “if-then” gives rise to only one from the outset (Byrne, 2005; Johnson-Laird & Byrne, 1989).

According to the model theory, illusions depend on initial models and not on fully explicit models, so it should be expected that whenever a conditional problem gives rise to a dual representation, as in the case of “only if” conditionals, the illusory response should be different from “if-then” conditionals that give rise to a single representation. One of our main research objectives was to test whether this prediction holds true. According to the model theory, when people read the conditionals “only if” (Illusory Example 2):
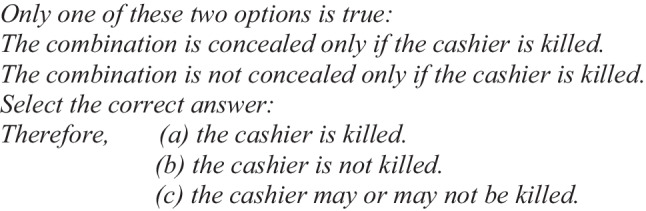


they build the following initial models:
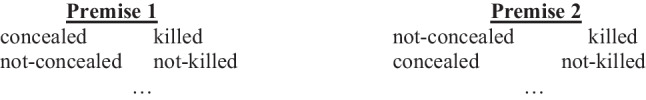


The above representation leads people to select the illusory option (c), “the cashier may or may not be killed,” more frequently than the other two options (a) or (b). This happens because people tend to make inferences from the initial models. As we can see, the mental models built from both “only if” premises present two possibilities, that “the cashier was killed” and “the cashier was not killed,” and participants should choose the illusory answer “the cashier may or may not be killed” as the correct one. Therefore, the model theory predicts that for “only if” conditionals (Illusory Example 2), participants should select the conclusion that “the cashier may or may not be killed” more frequently than “the cashier was killed” (see Table 1). However, for “if-then” conditionals (Illusory Example 1), participants should more frequently choose the conclusion “the cashier was killed” over the conclusion “the cashier may or may not be killed” (see Table 1).

The model theory predicts the same illusory response pattern for conditional “if-then” and biconditional “if and only if” problems because both problems have the same number of initial models, but unlike the other conditional expression, the biconditional has two fully explicit models instead of three. For example, the biconditional “if and only if the combination is concealed, then the cashier is killed” is represented by the two fully explicit models:
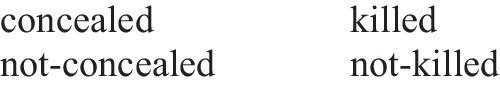


However, when people read the biconditionals “if and only if” (Illusory Example 3):
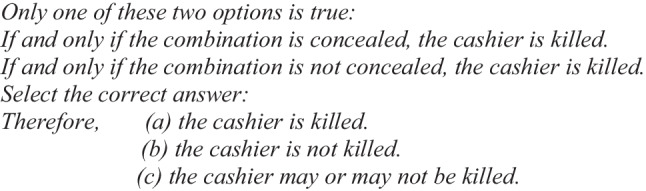


they build just the initial model (Johnson-Laird et al., 1992; Moreno-Ríos & Byrne, 2018):
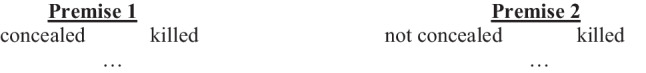


The above representation is equivalent to Illusory Example 1 (“if-then”). Consequently, a higher percentage of illusory responses will use “the cashier is killed” than “the cashier may or may not be killed” for “if-then” and “if and only if” problems. However, the correct answer for “if and only if” is “the cashier may or may not be killed.” To reach this conclusion, people need to flesh out the fully explicit models of both premises:
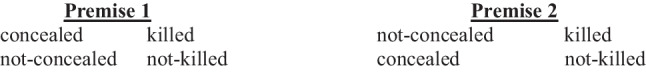


If participants consider that Premise 1 is true and Premise 2 is false, or vice versa, then they realize that the possibilities are contradictory. For example, the possibility “the combination is concealed, and the cashier is killed” is true for Premise 1 and false for Premise 2 (the same happens for the other possibilities). In either case, the correct conclusion is more likely for “if and only if” than it is for “if-then,” given that the latter answer demands more explicit models (three) than the biconditional (two) to get the correct answer. The greater the number of explicit models to be constructed, the harder the task should be; that is, the task should take longer, be more likely to lead to errors, and be rated as more difficult (Johnson-Laird et al., 1992).

## Experiment 1: Illusory inferences for “if-then” and “only if” conditionals

The aim of this experiment was to test the model theory’s prediction that an initial representation is responsible for an illusory response. To test this hypothesis, we used conditional problems with different initial representations (that is, single versus dual representation), but the same set of explicit models (three models; Johnson-Laird & Byrne, 1991). Also, to avoid some pragmatic effects we used conditionals with the same structure as in Illusory Example 1 and 2 but using arbitrary content applied in previous studies (e.g., Johnson-Laird & Savary, 1996). According to the model theory, Illusory Problem 1 (“if-then”) leads people to represent a single mental model of the premises, while Illusory Problem 2 (“only if”) leads people to represent two mental models of the premises.

To test these predictions, we also used control problems in which the initial representation corresponds to the correct response, so that it did not lead to illusory answers. Hence, the model theory’s predictions are as follows:

- there will be a higher percentage of illusory responses stating “there is an ace in the hand” than “there may or may not be an ace in the hand” for “if-then” problems;
- there will be a higher percentage of illusory answers stating “there may or may not be an ace in the hand” than “there is an ace in the hand” for “only if” problems;
- the percentage of correct answers should be similar for “if-then” and “only if” problems given the necessity of fleshing out the three explicit models;
- the percentage of correct answers should be higher for control problems than for illusory problems.

However, the suppositional theory does not explicitly differentiate predictions for the response patterns between both types of conditionals (“if-then” and “only if”). Specifically, we could expect that:

- there will be a higher percentage of illusory responses stating, “there is an ace in the hand” than responses stating, “there may or may not be an ace in the hand” for “if-then” and “only if” problems;
- the percentage of correct answers should be similar for “if-then” and “only if” problems.

### Method

#### Participants

Based on a previous study by Johnson-Laird and Savary (1999) and applying a binomial test and assuming an effect size of *g* = .2 (power .95 and alpha error of 5%), a minimum of 79 participants was needed for this experiment and subsequent experiments. At the National Distance Education University (UNED) in Spain, 80 undergraduates, all native Spanish speakers, participated in Experiment 1. They comprised 62 women and 18 men, whose ages ranged from 20 to 58 years and averaged 32 years. None of the participants of the three experiments had received instruction in logic, nor had they taken part in similar experiments in this research.

#### Design

The experiment consisted of a single design. Its independent variable was the problem type: “if-then” illusions, “only if” illusions, and control problems that made use of “and.” The experiment measured the type of conclusion participants chose (among three options: two determined and one undetermined) as well as their degree of confidence (on a scale between 1 and 5). The concept of “determined option” was selected for answers “a” (e.g., there is an ace in the hand) and “b” (e.g., there is no ace in the hand), and “undetermined option” for answer “c” (there may or may not be an ace in the hand).

#### Materials and procedure

Participants received four problems in their native Spanish language, two illusory and two control, and they were presented online using PsyToolkit (Stoet, 2010, 2017). No practice trial was presented. The instructions were:

*You will be given information about different cards from a deck of cards. You must take into account only the information indicated in the premises. Each problem consists of two premises, which appear on different lines. You must assume that one of the premises is true and the other false, but unfortunately you do not know which is true and which is false. Choose the correct conclusion.*

*You may take as long as you wish on each problem. Please complete the problems in the order they were given to you, and please do not go back to a problem to change your answer after you have completed it. For each problem you must also indicate your confidence in your conclusion. The degree of confidence ranges from “not confident” (1) to “very confident” (5). Indicate the number that reflects your degree of confidence.*

After the instructions, the problems were presented sequentially, and participants had to register their response to the problem in order to proceed to the next one. For the conditional “if-then” (Illusory Problem 1), an example was:
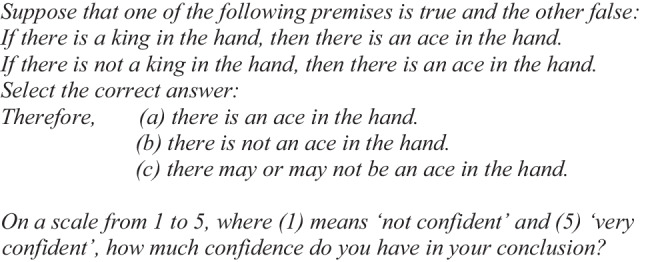


Participants were asked to select one of the three alternative responses. After that, they also had to choose in their response a degree of confidence on a Likert scale from 1 to 5. The same procedure was followed for all problems.

For the conditional “only if” (Illusory Problem 2), one example was the following:
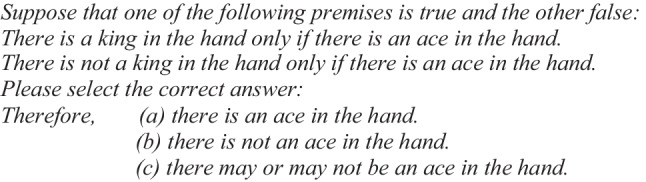


The correct answer in both illusory problems is “there is not an ace in the hand” because if the first conditional is false, then “there is a king in the hand and there is *not* an ace in the hand”; and if the second conditional is false, then “there is not a king in the hand and there is *not* an ace in the hand.” Either way, “there is not an ace in the hand.”

The control problems were two, for example:

Control Problem 1:
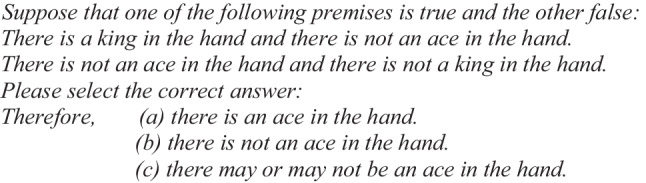


Control Problem 2:
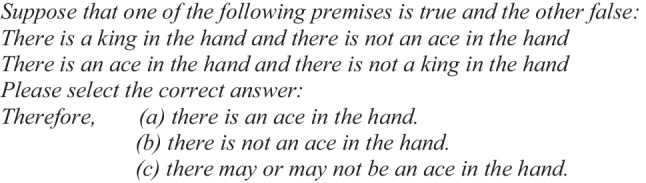


In Control Problem 1, the correct conclusion is the determined response (b), “there is not an ace in the hand.” This is because when the first premise is true, then “there is a king and there is not an ace,” and when the second premise is true, then “there is not an ace and there is not a king.” Either way, “there is not an ace.” In Control Problem 2, the correct conclusion is the undetermined conclusion (c), “there may or may not be an ace.” This is because when the first premise is true, then “there is a king and there is not an ace,” and when the second premise is true, then “there is an ace and there is not a king.” Illusory problems and control problems were presented at random. In each problem, a different type of content was used, always referring to the cards in a deck of cards (ace, king, queen, three, six, etc.). The content was counterbalanced among four different lists.

## Results and discussion

The data for this and subsequent experiments are available online via the Open Science Framework at: https://osf.io/pyv95/?view_only=31cd11c719314d97aa777349911bd110. Table 2 shows the percentages of responses given by the participants for each of the different options for illusory and control problems. First, for “if-then” problems, we found that the participants more frequently selected the option “there is an ace in the hand” than “there may or may not be an ace in the hand” (69% vs. 24%, Wilcoxon test, *z* = 4.19, *r* = .47, *p* < .001). Second, they chose the option “there may or may not be an ace in the hand” more than “there is an ace in the hand” for “only if” (64% vs. 31%, *z* = 2.98, *r* = .33, *p* = .005). Third, there were no differences in correct answers (“there is not an ace in the hand”) between “if-then” and “only if” problems (8% vs. 5%, *z* = .82, *r* = .09, *p* = .41). Fourth, there were more correct answers for the control than for the illusory problems (83% vs. 6%, *z* = 7.82, *r* = .87, *p* < .001).

**Table 2:** Percentages of responses for illusory (“if-then” vs. “only if”) and control problems in Experiment 1. The predicted conclusion according to the model theory appears in bold and the correct answer in italics according to formal logic

*Conclusions:*	Ace	not-ace	may /may not-ace
*Illusory problems:*			
“if-then”	**69**	*8*	24
“only if”	31	*5*	**64**
*Control problems:*			
King & not-ace/not-ace & not-king	7	***78***	15
King & not-ace/ace & not-king	5	7	***88***

Moreover, participants were confident in both their illusory and their control conclusions, which show no significant difference between them (3.9 vs. 4; *z* = .83; *r* = .09, *p* = .41). In addition, no differences were found between the particular “if-then” problem and the control problem with the determined conclusion (4.22 vs. 4.22; *z* = .31, *r* = .03, *p* = .76) nor between the “only if” problem and its control problem with the undetermined conclusion (3.60 vs. 3.73; *z* = .69, *r* = .08, *p* = .50). Results suggest that participants do not perceive illusory problems to be odder than control problems.

Experiment 1 showed that participants make different types of illusory inferences when they reason with “if-then” conditionals (e.g., if there is a king in the hand, then there is an ace in the hand) and when they reason with the same conditional but using the expression “only if” (e.g., there is a king in the hand only if there is an ace in the hand). These results conform to the predictions of the model theory but rule out the predictions of the suppositional theory. The model theory predicts, for the problems used in this research, a different pattern of outcomes for “if-then” than it does for “only if” conditionals (see Table 1). However, according to our predictions attributed to the suppositional theory, we would expect individuals to exhibit the same pattern of data when reasoning with these two types of conditionals (see Table 1). Also, the prediction of the model theory that the percentage of correct answers should be similar for “if-then” and “only if” problems was confirmed. This prediction is based on the idea that both problems require the three explicit models to reach the correct answer. Finally, the prediction of the model theory that the percentage of correct answers should be higher for control problems than for illusory problems was also confirmed. This prediction is based on the fact that the initial models in illusory problems lead people to the incorrect answer while the initial model in control problems leads people to the correct answer.

These findings are relevant not only because they confirm the main predictions of the model theory about illusions in conditional reasoning but also because the explanation given by the suppositional theory for illusions and the potential equivalence between expressions in conditional reasoning is experimentally questioned for the first time.

## Experiment 2: Illusory inferences for “if-then” conditional and “if and only if” biconditional

The aim of this experiment was to test the model theory’s prediction that the illusory response will be similar for the “if-then” conditional and the “if and only if” biconditional given they have the same initial representation, although they have different number of explicit models (that is, two models for the biconditional and three for the conditional “if-then”). We used the conditional “if-then” problem: “if there is a king, then there is an ace” or, if not, “if there is not a king, then there is an ace,” and the biconditional problem: “if and only if there is a king, then there is an ace” or, if not, “if and only if there is not a king, then there is an ace.” From the model theory, the conditional problem (“if-then”) and the biconditional problem (“if and only if”) lead people to represent a single mental model of the premises. The theory’s predictions are as follows:

- there will be a higher percentage of illusory responses that state, “there is an ace in the hand” than those that state, “there may or may not be an ace in the hand” for “if-then” and “if and only if” problems;
- the percentage of correct answers will be higher for “if and only if” problems than for “if-then” ones because the first demands two explicit models, and the second three explicit models, to reach the correct answer;
- the percentage of correct answers will be higher for the control problems than for the illusory problems.

Similarly, we could expect that the suppositional theory’s predictions posit that there will be the same response pattern for both types of conditionals (“if-then” and “if and only if”). Specifically:

- there will be a higher percentage of illusory responses stating, “there is an ace in the hand” than responses stating, “there may or may not be an ace in the hand” for “if-then” and “if and only if” problems;
- the percentage of correct answers will be similar for “if-then” and “if and only if” problems.

### Method

#### Participants

A different group of people from UNED participated in this experiment. The 79 volunteers, which included 61 women and 18 men, averaged 33 years in age, with a range from 19 to 62 years.

#### Design, materials, and procedure

The design, materials, and procedure for Experiment 2 were identical to Experiment 1, except that the conditional “only if” was replaced by the biconditional “if and only if.”

## Results and discussion

Table 3 shows the percentages of participant responses for each of the different options for illusory and control problems. We found that the participants selected the option “there is an ace in the hand” more frequently than “there may or may not be an ace in the hand” for “if-then” problems (56% vs. 34%, *z* = 2.01, *r* = .23, *p* = .045), but did not find significant differences between both options for “if and only if” problems (48% vs. 46%, *z* = .23, *r* = .03, *p* = .82). Also, participants chose the correct option for “if and only if” more frequently than “if-then” (46% vs. 10%, *z* = 4.32, *r* = .49, *p* < .001), while the percentage of correct answers for the control was higher than it was for the illusory problems (76% vs. 28%, *z* = 6.69, *r* = .75,* p* < .001). Finally, participants showed high confidence in both their illusory and their control conclusions (3.6 vs. 3.5; *z* = 1.14, *r* = .13, *p* =.25). Again, no significant differences were found between confidence judgments, for “if-then” and its determined control (3.76 vs. 3.63; *z* = .64, *r* = .07, *p* = .52) or for “if and only if” and its determined control (3.43 vs. 3.63; *z* = 1.26, *r* = .14, *p* = .21).

**Table 3:** Percentages of responses for illusory (“if-then” vs. “if and only if”) and control problems in Experiment 2. The predicted conclusion according to the model theory appears in bold and the correct answer in italics according to formal logic

*Conclusions:*	Ace	not-ace	may /may not-ace
*Illusory problems:*			
“if-then”	**56**	*10*	34
“if and only if”	**48**	6	*46*
*Control problems:*			
King & not-ace/not-ace & not-king	10	***70***	20
King & not-ace/ace & not-king	9	10	***81***

Experiment 2 showed that participants fall into different types of inferences when they reason with an “if-then” conditional (e.g., if there is a king in the hand, then there is an ace in the hand) and “if and only if” biconditional (e.g., if and only if there is a king in the hand, then there is an ace in the hand). These results run against the model theory and the equivalence between expressions attributed to the suppositional theory, which predict there should be the same pattern of illusory response for both types of problems. As we can see in Table 3, this prediction was not confirmed. People selected “there is an ace in the hand” more than they did “there may or may not be an ace in the hand” only for “if-then” problems as both theories predict, however people chose almost equally “there is an ace in the hand” and “there may or may not be an ace in the hand” for “if and only if” biconditional. A possible explanation will be given later in the general discussion. Also, we found that the correct answers were higher for “if and only if” problems than for “if-then” ones, as predicted by the model theory, and against the suppositional theory. And finally, the model theory prediction confirmed that the percentage of correct answers should be higher for control problems than for illusory problems.

## Experiment 3: Illusory inferences for “only if” conditional and “if and only if” biconditional

The aim of this experiment was to test the model theory’s prediction that the illusory response will be different for “only if” conditionals and “if and only if” biconditionals. According to the model theory, Illusory Problem 3 (“if and only if”) leads people to represent a single mental model of the premise, while Illusory Problem 2 (“only if”) leads people to represent two mental models of the premise. Moreover, they also have different numbers of explicit models (two models for the biconditional and three for the conditional “only if”). The theory’s predictions are as follows:

- a higher percentage of participants will choose the illusory answer “there may or may not be an ace in the hand” than will choose “there is an ace in the hand” for “only if” problems;
- a higher percentage of participants will choose the illusory answer “there is an ace in the hand” than “there may or may not be an ace in the hand” for “if and only if” problems;
- the percentage of correct answers will be higher for “if and only if” than for “only if” problems because the first demands two explicit models, and the second three explicit models, to reach the correct answer;
- the percentage of correct answers should be higher for the control problems than for the illusory problems.

However, according to the suppositional theory, we could expect that there will be the same response pattern for both types of conditionals (“only if” and “if and only if”). Specifically:

- there will be a higher percentage of illusory responses, “there is an ace in the hand” than responses “there may or may not be an ace in the hand” for “only if” and “if and only if” problems;
- the percentage of correct answers should be similar for “only if” and “if and only if” problems.

### Method

#### Participants

A different group of people from UNED participated in this experiment. The 80 volunteers, which included 61 women and 19 men, averaged 34 years in age, with a range from 19 to 80 years.

#### Design, materials, and procedure

The design, materials, and procedure in Experiment 3 were the same as those used for Experiment 1, with the exception that the conditional “if-then” was replaced by the biconditional “if and only if.”

## Results and discussion

Table 4 shows the response percentages given by the participants to each of the different options for illusory and control problems. We found that the participants selected the option “there may or may not be an ace in the hand” more frequently than they did for “there is an ace in the hand” in the case of “only if” problems (60% vs. 33%, *z* = 2.56, *r* = .29, *p* = .011), but there were no significant differences between both options for “if and only if” problems (41% vs. 53%, *z* = 1.03, *r* = .12, *p* = .30). Also, participants chose the correct option for “if and only if” more frequently than they did for “only if” (53% vs. 8%, *z* = 5.55, *r* = .62, *p* = < .001), and the percentage of correct answers was higher for the control than it was for the illusory problems (68% vs. 30%, *z* = 5.69, *r* = .64, *p* < .001). Finally, participants were confident in both their illusory and correct control conclusions without showing differences between them (3.62 vs. 3.64; *z* = .61, *r* = .07, *p* = .54). As, in the previous experiments, no significant differences were found between confidence judgments for “only if” and its undetermined control (3.58 vs. 3.45; *z* = .70, *r* = .08, *p* = .49) or for “if and only if” and its determined control (3.66 vs. 3.83; *z* = 1.17, *r* = .13, *p* = .24).

Experiment 3 replicated the results of Experiments 1 and 2. Participants tend towards different types of illusory inferences when they reason with “only if” conditionals (e.g., there is a king in the hand only if there is an ace in the hand) and “if and only if” biconditionals (e.g., if and only if there is a king in the hand, then there is an ace in the hand). These results challenge the equivalence between different conditional expressions attributed to the suppositional theory, which predicts that people should select the response “there is an ace in the hand” more than “there may or may not be an ace in the hand” for both types of problems. As we can see in Table 4, this prediction was not confirmed. However, these results only partially confirm the predictions of the model theory. According to this, there should be a higher percentage of illusory responses saying “there may or may not be an ace in the hand” than “there is an ace in the hand” for “only if” conditionals, as Table 4 confirms. Moreover, the model theory predicts that there should be a higher percentage of illusory responses saying “there is an ace in the hand” than “there may or may not be an ace in the hand” for “if and only if” conditionals. However, people chose equally both responses, namely, “there is an ace in the hand” and “there may or may not be an ace in the hand” (see Table 4). A possible explanation will be given later in the general discussion.

**Table 4:** Percentages of responses for illusory (“only if” vs. “if and only if”) and control problems in Experiment 3. The predicted conclusion according to the model theory appears in bold and the correct answer in italics according to formal logic

*Conclusions:*	Ace	not-ace	may /may not-ace
*Illusory problems:*			
“only if”	33	*8*	**60**
“if and only if”	**41**	6	*53*
*Control problems:*			
King & not-ace/not-ace & not-king	7	***64***	29
King & not-ace/ace & not-king	14	14	***73***

Also, we found that the correct answers were higher for “if and only if” than for “only if” problems, as predicted by the model theory (e.g., Johnson-Laird et al., 1992), and against the suppositional theory predictions. Finally, the model theory prediction that the percentage of correct answers should be higher for control problems than for illusory problems was confirmed.

## General discussion

Most people are aware of their cognitive limitations in daily life activities. We know that even if we pay full attention, using all our attentional resources, the result will be the same. However, demonstrations of the illusory responses in deduction are not so frequent, and we would expect that people will try to look for possible explanations instead of questioning the reliability of their own deductive systems. In the previous examples of the cashier and the card studies, people tend to conclude the opposite of what is valid. In this research, we do not need to test validity based on formal logic. Instead, we can assume the concept of validity based on Jeffrey’s (1981) proposal that an inference is valid if the conclusion is true in each case in which the premises are also true. Illusory conclusions were first demonstrated by Johnson-Laird and their collaborators (see review by Khemlani & Johnson-Laird, 2017), and they were shown to exhibit different kinds of relationships such as “if-then” conditionals (Johnson-Laird & Savary, 1999), co-references (e.g., Koralus & Mascarenhas, 2013), set members (e.g., Santamaria & Johnson-Laird, 2000), causal relations (e.g., Goldvarg & Johnson-Laird, 2000), spatial relations (e.g., Ragni et al., 2016), and deontic reasoning (Bucciarelli & Johnson-Laird, 2005).

The main aim of this research was to study the roles that different expressions of conditionals (“if-then,” “only if,” and “if and only if”) play in the illusory response. We consider illusory responses to be those wrong answers that individuals systematically give with high confidence to specific problems. Experiment 1 showed that for problems with a single representation (“if-then”), participants preferred the illusory response “there is an ace in the hand,” while for problems with two initial representations (“only if”), participants chose the illusory response “there may or may not be an ace in the hand.” These results are in accordance with the predictions of the model theory, which argues that illusions in reasoning occur because people reason from the initial representation of true possibilities (Johnson-Laird & Savary, 1999; Khemlani & Johnson-Laird, 2017), but not with the predictions of the suppositional theory (Evans & Over, 2004; Evans et al., 2005). This latter theory explains that these results in illusory problems “if-then” are due to participant failure to apply the exclusive disjunction between the conditional premises, based on the assumption that a conditional can be true only when the antecedent is true. It is important to note that the theory only makes explicit predictions about illusory problems with “if-then” but not with other conditional expressions, and therefore prediction with other expressions are speculative. We did not find reasons to make different predictions from this theory when the illusory problems were constructed using other conditional expressions. Because the conditional “if-then” is equivalent to the conditional “only if,” we assumed that this theory would predict the same pattern of data for both types of conditionals: participants should select more frequently the conclusion “there is an ace” for both conditional problems. Nonetheless, Experiment 1 did not support this prediction. In brief, Experiment 1 not only confirms the main predictions of the model theory about illusions in conditional reasoning but also, for first time, experimentally questions the potential explanation of equivalence between expressions attributed to the suppositional theory for illusions in conditional reasoning.

Therefore, we have replicated previous studies with “if-then,” tested the explanation given by the model theory, and contrasted it with the suppositional theory using different conditional expressions. If the illusion was due to the initial models, as the model theory proposes, then other expressions with the same initial models should produce similar illusions, except those with different sets of initial models. In previous studies (Byrne, 2005; Espino & Byrne, 2013; Espino & Santamaría, 2008; Johnson &-Laird & Byrne, 1989; Santamaría & Espino, 2002; Thompson & Byrne, 2002), it was demonstrated that “only if” conditionals were represented with two different initial models, while “if-then” were represented by only one.

In Experiment 2, we again used “if-then” conditionals and paired them with “if and only if,” another common conditional expression that is represented with one initial model. Therefore, the prediction was that illusory conclusions should be similar for both conditionals. However, Experiment 2 showed that participants were more likely to use different types of illusory inferences whenever they reasoned with “if-then” conditionals (e.g., if there is a king in the hand, then there is an ace in the hand) and when they reasoned with “if and only if” biconditionals (e.g., if and only if there is a king in the hand, there is an ace in the hand). In the case of results for “if-then” problems that support Experiment 1, participants selected the option “there is an ace” more frequently than they did the options “there is not an ace” or “there may or may not be an ace.” However, for “if and only if” problems, there was not a systematic response as would be expected for an illusory response. Participants selected the option “there may or may not be an ace” as frequently as the option “there is an ace.” That is, the patterns of conclusions differed between the two conditionals.

These results do not confirm the model theory’s prediction that illusory inferences will also happen with biconditional expressions. Now, the model theory can explain the difference in responses between the two conditionals if we take into account implicit models. The reason for this is that some people may incorporate some of the implicit models (e.g., “not-king and not-ace”) into the initial representation. There is greater probability to include the model “not-king and not-ace” in the initial representation for the biconditional “if and only if there is a king in the hand, then there is an ace” than there is for the conditional “if there is a king in the hand, then there is an ace” (see Johnson-Laird et al., 1992; Moreno-Ríos & Byrne, 2018). The inclusion of this fleshed-out model in the representation leads participants to accept option (c) more frequently in biconditionals than in “if-then” conditionals.

Experiment 3 replicated the previous experiments. As in Experiment 1, for “only if” problems, participants selected the option “there may or may not be an ace” more frequently than other options (such as “there is an ace” or “there is not an ace”). As in Experiment 2, for “if and only if” problems, participants selected the option “there may or may not be an ace” as frequently as they did “there is an ace.” These results are due to the fleshed-out model. Again, the systematicity in one response, characteristic of illusory responses, is only present in “only if” conditionals but not in “if and only if” conditionals given that both responses (e.g., “there may or may not be an ace” and “there is an ace”) were chosen similarly.

Below, we present some alternative explanations to those offered by the model theory. However, we show that none of them is adequate to explain the data obtained in the three experiments. The first alternative explanation is that the illusory response is due to matching bias (Evans, 1975; Evans & Newstead, 1977). In this bias, people prefer to mentally represent the items mentioned in the premises. For example, when people read the premises “if there is a king, then there is an ace” and “if there is no king, then there is an ace,” they represent the card “there is an ace” for both the first and the second premises. Thus, people would conclude that “there is an ace” since this card is presented in both premises. The matching bias is compatible with the data obtained in our research using the conditional “if-then” (Experiments 1 and 2) but not with the conditional “only if” (Experiments 1 and 3). In this type of problem, participants selected the response “there may or may not be an ace” more frequently than “there is an ace.” Matching bias predicts that the most frequent response should be “there is an ace” for all expressions. Finally, matching bias does not explain the pattern of data obtained through biconditionals (Experiments 2 and 3). Participants selected the responses “there is an ace” as well as “there may or may not be an ace.” In summary, our experiments excluded the possibility that the illusory response can be explained as a matching effect.

The second alternative explanation is that the illusory response resulted from the fact that the task, instructions, or premises of illusions are perceived as so complex or ambiguous that participants are confused, and as a result commit fallacies (e.g., Johnson-Laird & Savary, 1999; Khemlani & Johnson-Laird, 2017). Following previous decisions to rule out this possibility (De Neys et al., 2013, p. 269; Khemlani & Johnson-Laird, 2017, p. 25), we had to discard the possibility that illusory problems are not perceived as more difficult than control problems, resulting in lower confidence values. The results do not support this alternative explanation. Experiments 1, 2, and 3 showed that participants were highly confident in both their illusory conclusions and valid control conclusions, albeit without displaying a reliable difference between confidence ratings.

This study examines whether the same illusory response observed in “if-then” conditionals also occurs in other conditional expressions. There are two important aspects related to the concept of “illusions”: they must be incorrect and systematic, and people must be confident in them. The results were consistent with illusory responses for “if-then” and for “only if” conditionals, although these responses differed. People selected “there is an ace” for “if-then” conditionals, while they chose “there may or may not be an ace” for “only if” conditionals. Additionally, they also showed that “if and only if” conditionals did not elicit a uniquely preferred response from participants. Therefore, we think that incorrect conclusions for the two first conditionals, but not for the third, should be referred to as “illusions.” We also assessed the participants” level of confidence in their incorrect conclusions and found that it was not lower compared to control problems.

Using the degree of confidence measurement is based on the logic that if illusory problems are perceived as more difficult than control problems, participants should exhibit lower confidence in the former (see Khemlani & Johnson-Laird, 2017). Effortful thought is expected to be accompanied by lower confidence than easy thought, even when the latter can be incorrect (Ackerman & Zalmanov, 2012). Similarly, lower confidence would be expected if participants were guessing about illusory problems rather than control problems. Other studies have shown that participants rated well-known illusory problems with low confidence, although they were assumed to require automatic processing (De Neys et al., 2013, with the bat-and-ball problems). While the confidence ratings obtained in the present experiments were high and did not differ from control problems, we must be cautious in interpreting the possible link between high confidence and automatic processing.

Finally, there are some other limitations to this study. Fallacies are presented as logical arguments, but we assume that people think similarly in other, more everyday ecological situations. The reason, like most reasoning studies, is to have better control over other intervenient spurious variables. Moreover, we believe it would be intriguing to test illusory problems using different content types, such as causal and temporal relationships, to expand the testing scope and evaluate reasoning theories. Also, we assume that the same factors tested here would apply to other kinds of conditionals. This has not been tested yet, but it could be interesting to test predictions using the conditional expressions “unless” or “even if.” We selected “if-then,” “only if,” and “if and only if” because they are very frequent in both everyday life and reasoning studies, and we have independent studies with empirical evidence of their representations, their difficulty, and their mutual comparison.

In sum, a novel aspect of this research is its demonstration that the role of the initial representation of the premises is a key factor that enhances the illusions, while the fleshed-out model weakens them. In fact, the fewer the number of fully explicit models, the fewer the number of illusions. These results are consistent with the idea that illusions in reasoning are a real phenomenon due to people’s tendency to represent the true initial possibilities of statements (Johnson-Laird, 2006; Khemlani & Johnson-Laird, 2017). Another relevant contribution of this research is the provision, for the first time, of experimental evidence that questions the potential equivalence between conditional expressions in the illusion and the necessity to account for this fact in other theories such as the suppositional theory.
